# A novel multi-task machine learning classifier for rare disease patterning using cardiac strain imaging data

**DOI:** 10.1038/s41598-024-61201-4

**Published:** 2024-05-09

**Authors:** Nanda K. Siva, Yashbir Singh, Quincy A. Hathaway, Partho P. Sengupta, Naveena Yanamala

**Affiliations:** 1 School of Medicine, West Virginia University, Morgantown, WV USA; 2 Division of Cardiology, Heart and Vascular Institute, West Virginia University, Morgantown, WV USA; 3 Department of Radiology, Mayo Clinic, Rochester, MN USA; 4 Division of Cardiovascular Disease and Hypertension, Rutgers Robert Wood Johnson Medical School, 125 Patterson St, New Brunswick, NJ 08901 USA; 5 Institute for Software Research, School of Computer Science, Carnegie Mellon University, Pittsburgh, PA USA

**Keywords:** Echocardiography, Machine learning, Constrictive pericarditis, Restrictive cardiomyopathy, Rare disease, Computational biology and bioinformatics, Cardiology, Medical research, Engineering, Mathematics and computing

## Abstract

To provide accurate predictions, current machine learning-based solutions require large, manually labeled training datasets. We implement persistent homology (PH), a topological tool for studying the pattern of data, to analyze echocardiography-based strain data and differentiate between rare diseases like constrictive pericarditis (CP) and restrictive cardiomyopathy (RCM). Patient population (retrospectively registered) included those presenting with heart failure due to CP (n = 51), RCM (n = 47), and patients without heart failure symptoms (n = 53). Longitudinal, radial, and circumferential strains/strain rates for left ventricular segments were processed into topological feature vectors using Machine learning PH workflow. In differentiating CP and RCM, the PH workflow model had a ROC AUC of 0.94 (Sensitivity = 92%, Specificity = 81%), compared with the GLS model AUC of 0.69 (Sensitivity = 65%, Specificity = 66%). In differentiating between all three conditions, the PH workflow model had an AUC of 0.83 (Sensitivity = 68%, Specificity = 84%), compared with the GLS model AUC of 0.68 (Sensitivity = 52% and Specificity = 76%). By employing persistent homology to differentiate the “pattern” of cardiac deformations, our machine-learning approach provides reasonable accuracy when evaluating small datasets and aids in understanding and visualizing patterns of cardiac imaging data in clinically challenging disease states.

## Introduction

Effectively interpreting the vast amount of medical data generated daily in healthcare is paramount to improving clinical decision-making. Machine learning has been employed in the healthcare field to discover meaningful trends. For cardiac imaging, advances in artificial intelligence have improved both the speed and accuracy of image interpretation as well as have facilitated detection of subtle changes in cardiac structure and function. Many studies have demonstrated the value of artificial intelligence in cardiac imaging, deep learning to classify left ventricular hypertrophy in echocardiography images^1^, structured random forests to trace myocardium borders in 3D echocardiograph volumes^2^, and convolution neural network model to develop high resolution images from 2D magnetic resonance image stacks^3^.

However, the large number of attributes present in many datasets must be reduced to prevent overwhelming the machine learning algorithms. For example, data derived from cardiac deformation analyses capture left ventricular wall motion in both 2- and 3-dimensional planes, generating a significant amount of data for machine learning applications^4^. Although we have a vast amount of data, today’s clinical evaluations usually depend on just one data point. The methods we use to gain insights from all the data haven’t shown to be more helpful in guiding patient care^5^.

Dimensionality reduction techniques, such as Principal Component Analysis and Linear Discriminant Analysis, are commonly applied techniques to better aggregate large feature sets. Another dimensionality reduction technique growing in prominence is Topological Data Analysis (TDA), which extracts data features based on local geometry and global topology encoded in the distribution of data points.

Many studies have incorporated general TDA principles in identifying patterns in nature, e.g., zebrafish pattern variability^6^, stability of protein folding^7^, and outcomes in preclinical traumatic brain injury and spinal cord injury^8^. Other groups have specifically utilized Persistent Homology from the TDA toolbox to understand human anatomy and physiology, e.g., to study brain artery branching and looping^9^, gait signals in patients with neurodegenerative diseases^10^, and MRI liver images in patients with Primary Sclerosing Cholangitis^5^. In this study, we propose a workflow for applying persistent homology to study high-dimensional data where the ratio of sample to features is very low.

**Figure 1:**
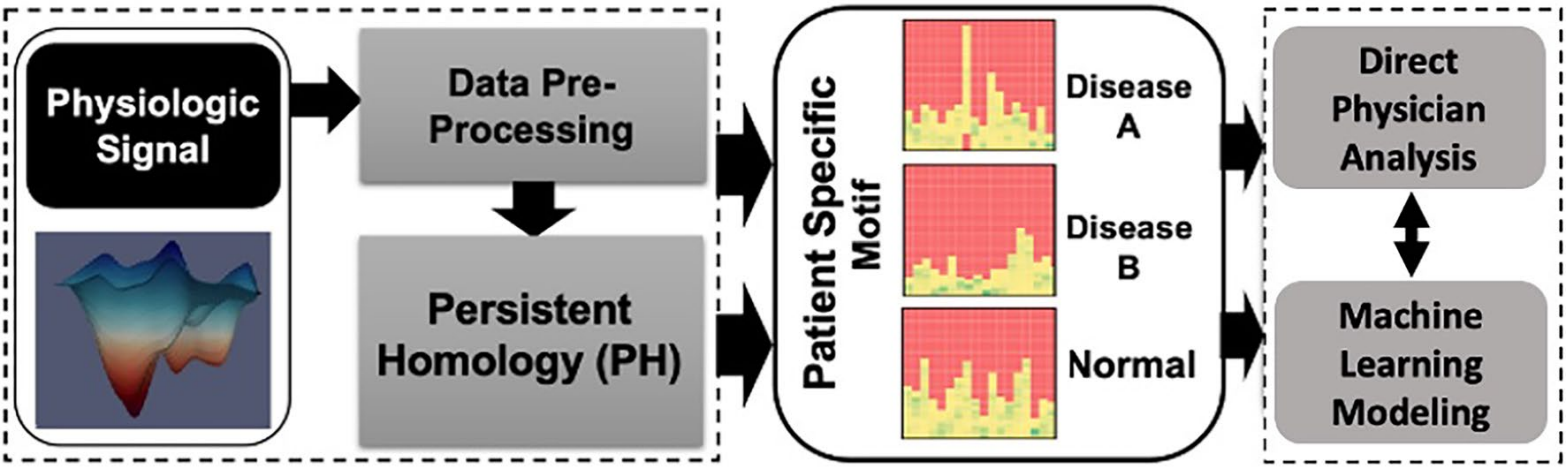
Outline of the general proposed workflow. Physiological signal is pre-processed to a suitable input for persistent homology (PH) feature extraction. The resulting topological features are represented visually as an individualized patient motif produced by concatenating multiple persistent images. This motif can be directly interpreted by physician or used to develop machine learning models.

**Figure 2:**
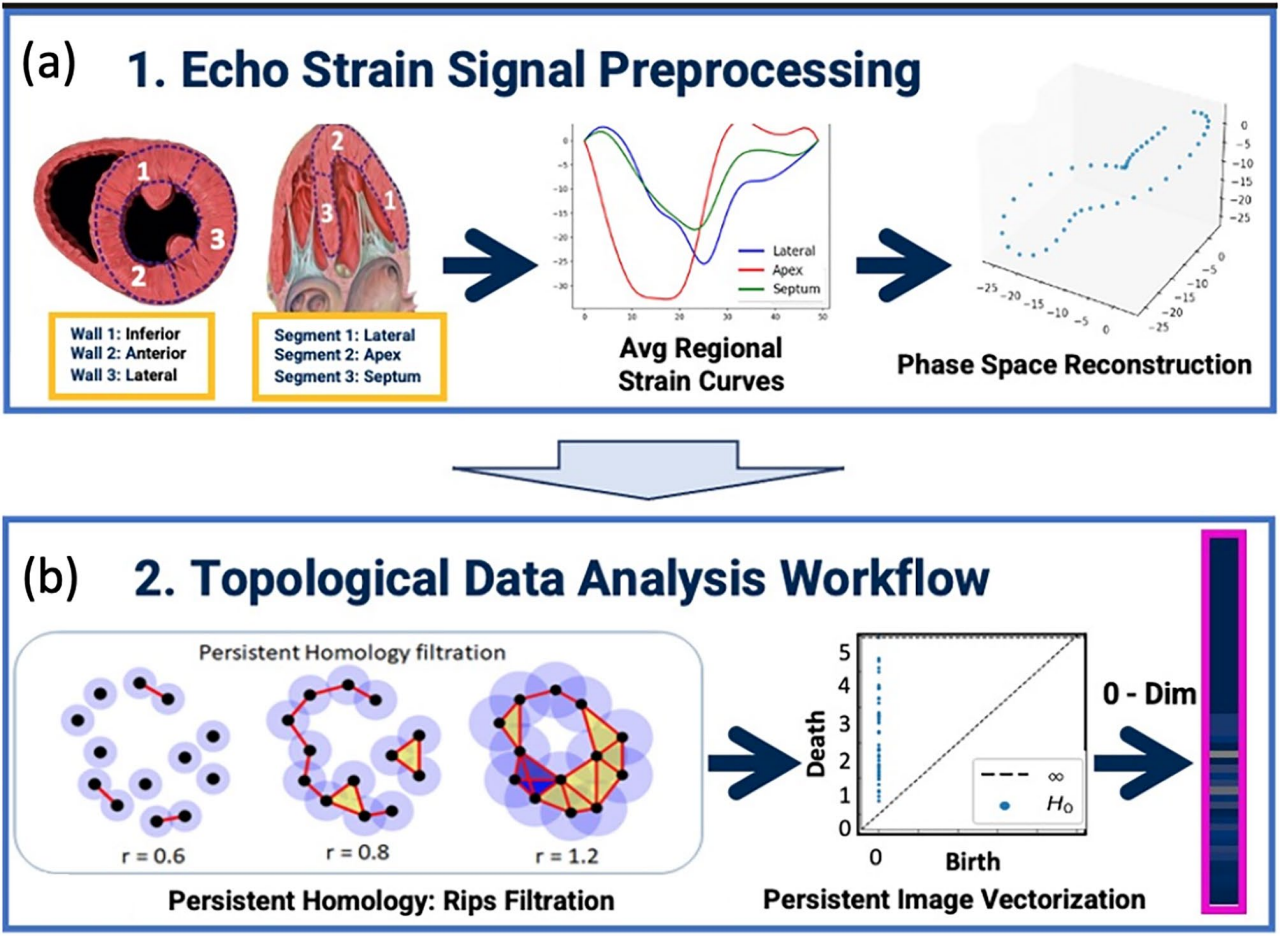
Schematic overview of use case scenario. Three regions were defined in the mid short axis and apical four chamber view; the average regional curve for each strain parameter was calculated and transformed using phase space reconstruction (**a**). This served as the input for persistent homology filtration; the resulting birth and death coordinates, based on radius (r) values, for dimension 0 were converted to a feature vector form for machine learning using persistent image methodology (**b**).

**Figure 3:**
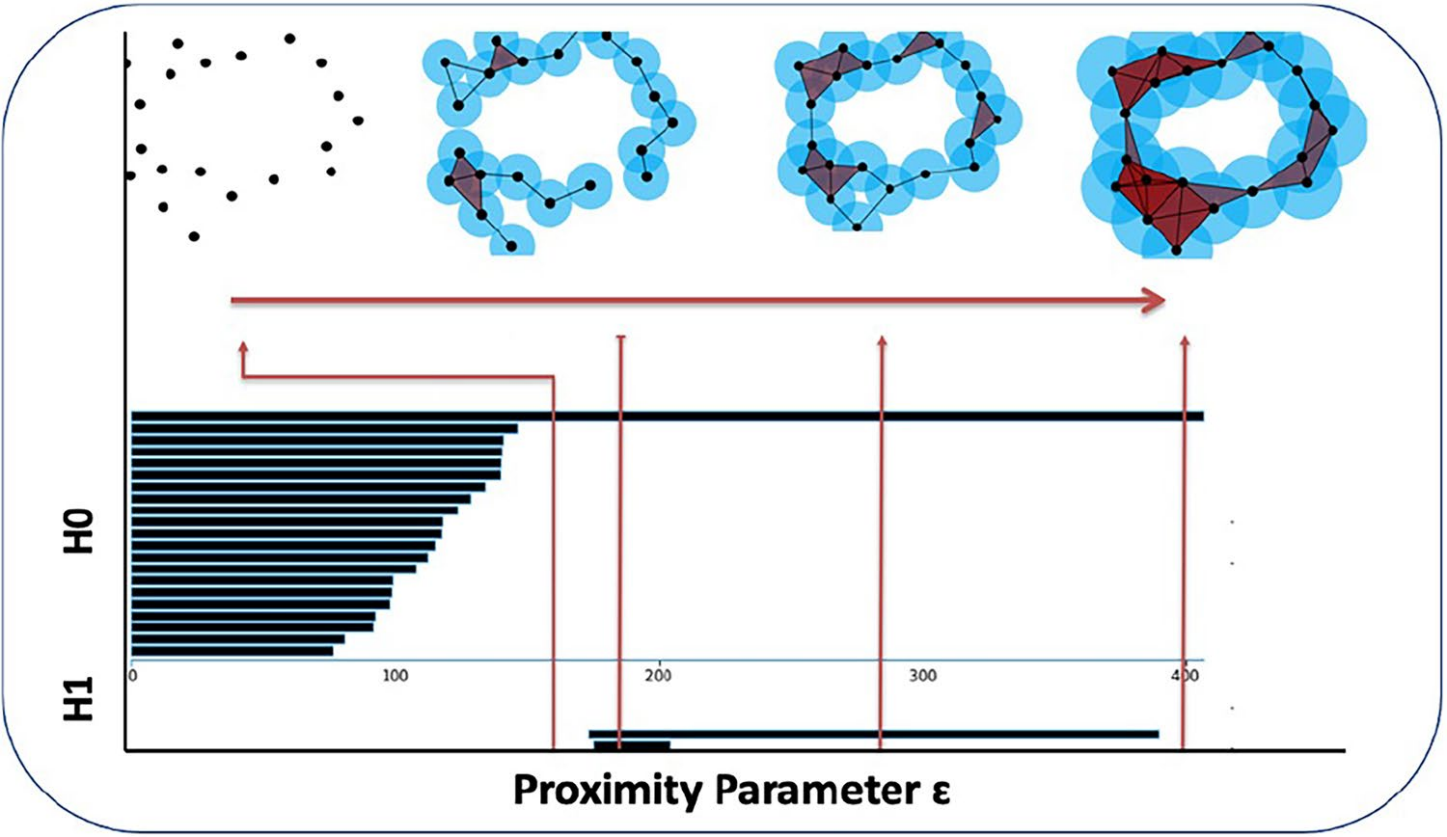
Persistent Homology Explanation**.** For a given proximity parameter ε, a circle with radius = ε is drawn around each data point. The intersections of these circles guide the construction of a set of simplicial complexes. All possible values of ε are tested to detect variations in topology at different scales. The appearance and disappearance of connected components and open loops is measured by H0 and H1, respectively, and subsequently visualized as a persistent barcode. H0 represents dimension 0 persistent homology, and H1 represents dimension 1 persistent homology.

The purpose of this study is to determine if topological data analysis can help in identifying patterns in echocardiography images to improve diagnostic accuracy for rare cardiac conditions. We provide a use-case scenario of our workflow that harnesses both deformation patterns and global topological information of the left ventricle to characterize cardiovascular diseases (Figs. 1, 2, 3).

## Results

### Average strain pattern motifs

**Figure 4:**
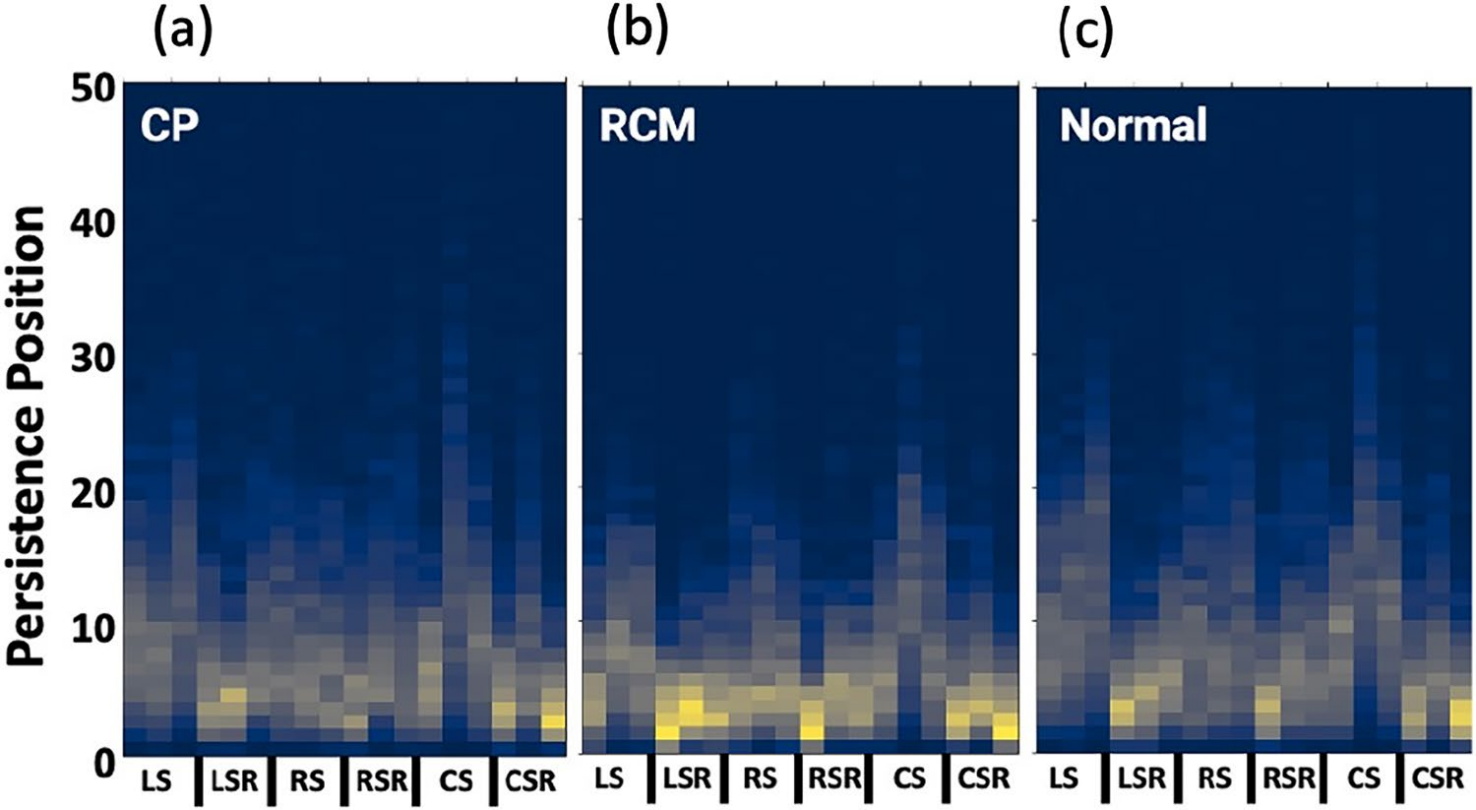
Cardiac condition average strain motifs. The output visual signature for persistent homology workflow was a heatmap-like motif with x-axis corresponding to strain or strain rate in longitudinal, radial, and circumferential directions; y-axis corresponding to persistence pixel position in the resulting 1 by 50 vector for each combination of wall region and strain measurement; and z-color corresponding to the pixel intensity calculated through persistent image vectorization. The average strain motifs for constrictive pericarditis, restrictive cardiomyopathy, and normal/control patients are shown to demonstrate group defining patterns. *CS* circumferential strain, *CSR* circumferential strain rate, *CP* constrictive pericarditis, *LS* longitudinal strain, *LSR* longitudinal strain rate, *RCM* restrictive cardiomyopathy, *RS* radial strain, and *RSR* radial strain rate.

The average strain patterns motifs for each cardiac condition are shown (Fig. 4). From visual inspection, general trends can be identified, e.g., RCM patients generally have higher intensity values limited to lower persistence pixels while having lower intensity values at higher persistence pixels. This indicates a restrictive pattern within these patients as it suggests that a fully connected component in the H0 dimension is formed at a lower scale parameter. Alternatively, for both CP and normal groups, the average motifs showed a much wider spread in their strain persistence pixel intensities, indicating more spacing in these patients’ data points than the general constraint seen in RCM.

### Feature selection

While the number of features was considerably reduced from 14,700 to 900 (18 by 50), this data still exhibited a high feature to sample ratio. The selected features through Boruta feature selection were used to develop predictive models for distinguishing between CP, RCM, and normal patients.

### Machine learning classifiers

**Figure 5:**
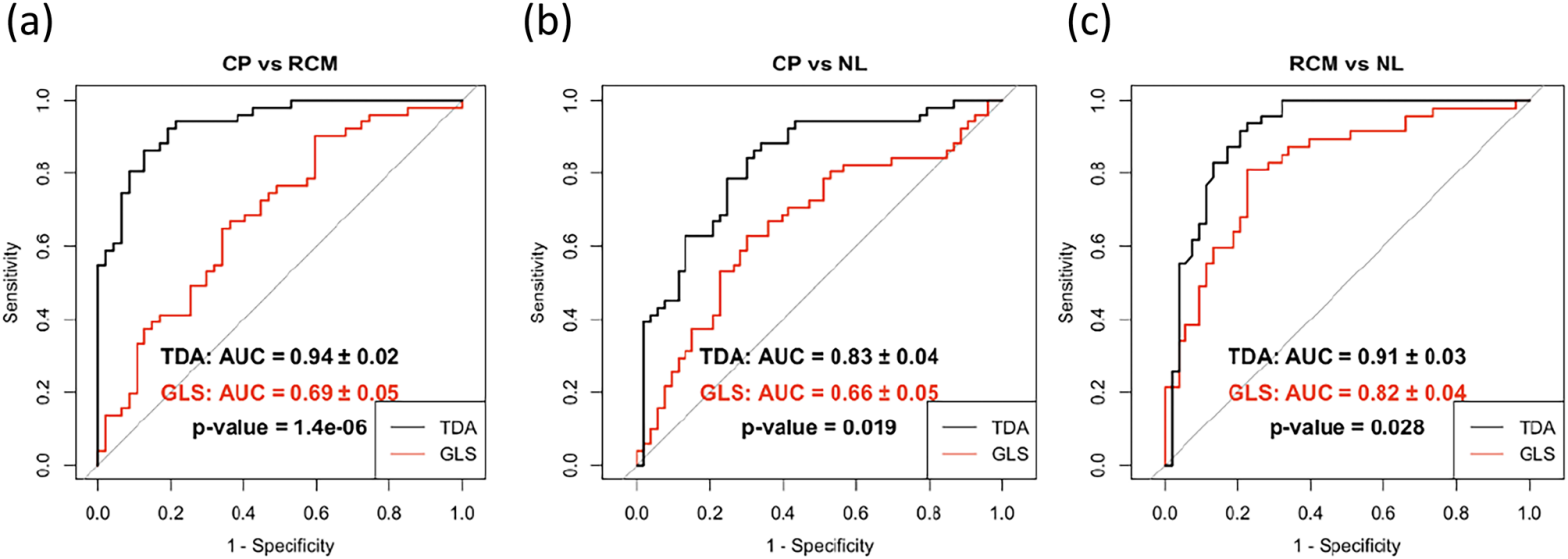
Binary classifier receiver operating curves. Cross-validation (tenfold) area under the curve (AUC) and respective p values for the models classifying binary conditions, restrictive cardiomyopathy (RCM), Constrictive pericarditis (CP), and normal (NL) is shown. Across all binary class distinctions, the persistent homology workflow outperformed the performance metrics of the GLS model. *GLS* Global Longitudinal Strain.

To determine if the features extracted through our pipeline helped distinguish the cardiac conditions, we developed three binary class classifiers for CP vs. RCM, CP vs. normal, and RCM vs. normal. The combined dataset from Amaki et al.^11^ and Sengupta et al.^12^ was evaluated using tenfold cross-validation. Finally, we compared the performance of these models with a baseline performance achieved by logistic regression models using average peak longitudinal strain from the 4Ch view. This peak value approximates the global longitudinal strain that clinicians typically extract from cardiac strain imaging data (Fig. 5).

Our CP vs RCM logistic regression classifier showed a statistically significant improvement compared to the GLS model (PH AUC = 0.94; GLS AUC = 0.69; p = 1.4 × 10^–6^). Our CP vs normal logistic regression classifier demonstrated improvement as well (PH AUC = 0.83; GLS AUC = 0.66; p = 0.019). Our RCM vs normal random forest classifier showed a statistically significant improvement compared to the GLS model (PH AUC = 0.91; GLS AUC = 0.82; p = 0.028). We created a multi-class random forest classifier to discriminate between all conditions; the average across all classes AUC, sensitivity (Sn), and specificity (Sp) were improved in comparison to the baseline model. Our PH model achieved AUC = 0.83 (Sn = 68% and Sp = 84%) whereas GLS model achieved AUC = 0.68 (Sn = 52% and Sp = 76%).

### Interpretable artificial intelligence

**Figure 6:**
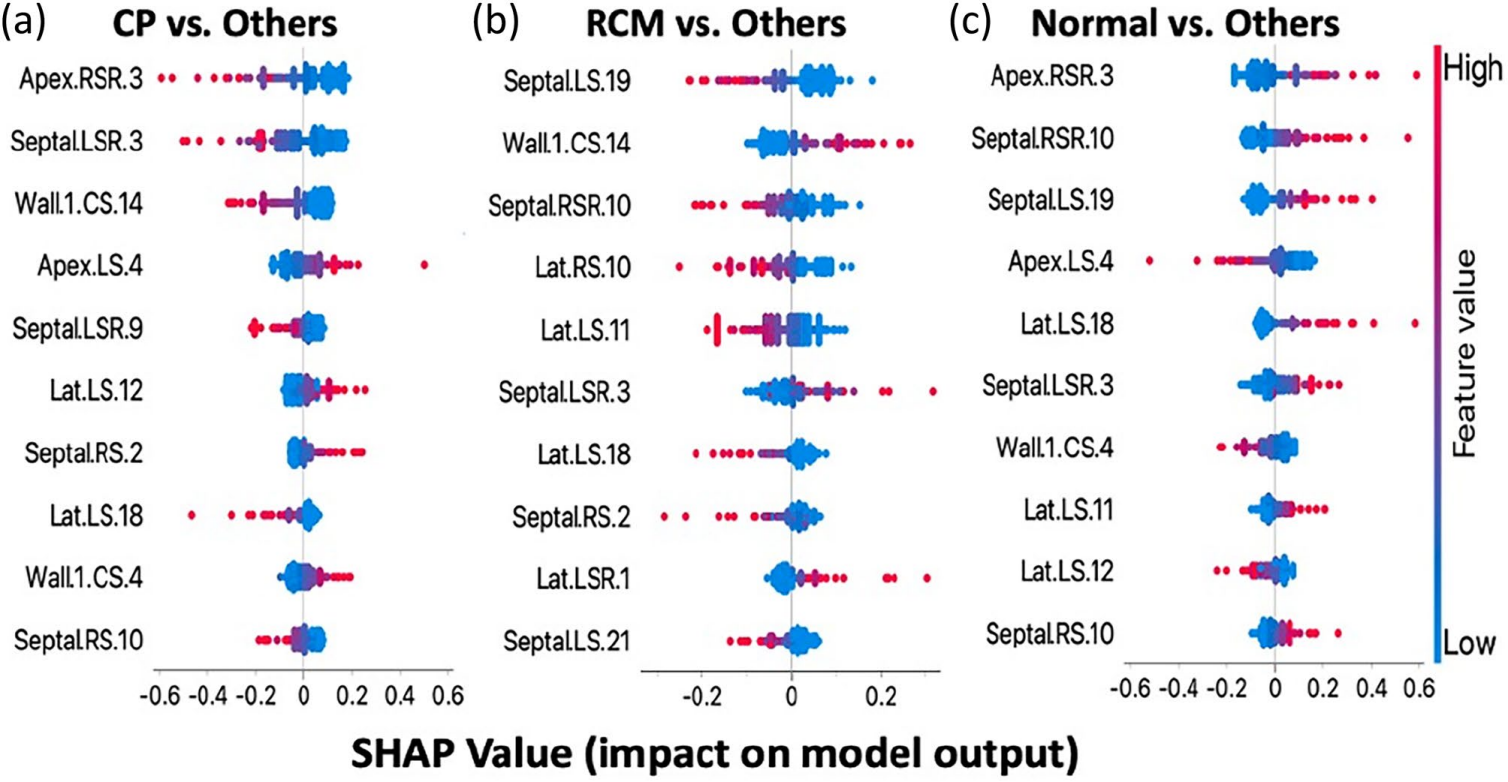
Shapley additive explanations for multi-class model. Shapley plot presents the top ten important features responsible for the multi-class machine learning model to output its predictions that discriminated each cardiac condition from the other two, i.e., constrictive pericarditis (CP) from restrictive cardiomyopathy (RCM) and Normal. The features identified from this interpretable artificial intelligence tool can be corresponded to specific regions in the patient specific motif that contribute to disease stratification visually. *CS* circumferential strain, *CSR* circumferential strain rate, *lat* lateral wall, *LS* longitudinal strain, *LSR* longitudinal strain rate, *RS* radial strain, and *RSR* radial strain rate.

We show the interpretable artificial intelligence results as Shapley additive explanation plots indicating the top ten features integral in distinguishing each class from the others (Fig. 6). Thus, a combination of feature trends is responsible for the model to output a particular prediction. Moreover, these results allow better comprehension of the average strain motifs produced (Fig. 4). To understand these patterns, we can refer to the original phase space reconstruction point clouds for septal longitudinal strain; for convenience, a few example patients from each disease group are depicted in Supplementary Figs. 1 and 2.

## Discussion

Compared with traditional echocardiography approaches, our methodology uses subclinical features identified using topological data extraction of segmental strain analysis to more clearly delineate clinically similar cardiac phenogroups, such as restrictive cardiomyopathy (RCM) and constrictive pericarditis (CP). Global and local structural deformations of the cardiac myocardium were captured with persistent homology, enabling us to predict the presence of CP (AUC: 0.83) and RCM (AUC: 0.91) from normal patients. Additionally, when directly differentiating between the two types of heart failure, CP and RCM (AUC: 0.94), our model demonstrated a comparable combination of sensitivity (92%) and specificity (81%) compared to the sensitivity (87%) and specificity (91%) shown in a study by Welch et al. at the Mayo Clinic investigating the conventional evaluation of constrictive pericarditis from restrictive myocardial disease or severe tricuspid regurgitation based on five principal echocardiographic features, including respiration related ventricular septal shift, maintained or greater medial mitral annular e′ velocity, and expiratory diastolic reversal ratio of the hepatic vein^13^. To provide context of our workflow’s comparison to another non-invasive method assessing clinically similar cardiac phenogroups, in a study by Masui et al. cardiac MRI was used to differentiate CP from RCM, with a sensitivity of 88% and specificity of 100%; our model performed worse in terms of specificity but better in terms of sensitivity^14^. While our TDA/echocardiography model performs similarly, it is important to note that ultrasound-based techniques offer a wider range of accessibility to patients than either CT or MRI.

In this small cohort, we highlight the ability of our TDA model to make accurate predictions of rare disease presentations as a use-case example for future cardiovascular applications. In diseases with low prevalence, this presents an obvious advantage to traditional approaches that may require a specific threshold of cases before allowing appropriate stratification. Focal involvement, either decompensation or compensation, of the myocardium will be captured in our current workflow in the appropriate wall region which would be useful in both common and rare cardiac conditions, such as myocardial infarction and apical hypertrophic cardiomyopathy, respectively. However, in order to gain more granularity to exactly which portion the deviation originates from, our workflow could subgroup the left ventricular wall into the American Heart Association 18–19 segments^15^, which would also require expanding our patient specific motif.

Previous investigations have endeavored to apply segmental strain analysis to predict structural and/or functional outcomes in the heart. Tabassian et al. used principal component analysis to represent the complex spatio-temporal nature of stress–strain curves and utilized machine learning to classify patients with myocardial infarctions^16^. Senapati et al. proposed a relative regional strain ratio (a metric of relative longitudinal strain sparing in the apex) to provide prognostic information in cardiac amyloidosis patients^17^. While these applications provide meaningful interpretation of segmental strain data, their translation to diverse cardiac phenotypes is likely limited by the lack of integration of the ultrastructural component of the left ventricle (LV). Another limitation of segmental strain data is high variability in measurements between vendors^18^; we attempt to reduce the effects of this issue in our workflow through standardizing patients’ cardiac cycles with spline interpolation and by aggregating individual segments into larger functional wall regions. The EACVI-ASE Strain Standardization Task Force recommends utilizing segmental strain pattern analysis rather than single segmental strain values^19^. We believe application of our protocol inherently accomplishes this as persistent homology is a topological data analysis tool that describes the shape of data by extracting its topological invariants^20^.

TDA has been increasingly applied to areas of biomedical research^10,21,22^ but only recently been evaluated in cardiovascular medicine. Specifically, diagnostic tests such as the ECG have provided the first applications of TDA in converting simple waveforms to numeric data^23,24^. More recently, TDA has been proposed as a method for the assessment of vascular diseases^25^ and has even provided improved predictive capacity for detecting acute coronary syndrome or revascularization in patients with coronary plaques than through the use of more commonly used clinical markers, such as risk factors, stenosis, and high-risk plaque features^26^.

Our work can be translated into clinical applications such as a medical decision support system for physicians, AI virtual assistant for patients, or an automated image analysis software. To facilitate accessibility for physicians and scientists with varying levels of expertise/understanding, we plan to provide the visual motifs with annotations and labels; providing comparison images of disease states and normal motifs will also enhance comprehension for a broader audience.

The limitation inherent to many studies investigating rare diseases is the relatively small sample size, which can be due to practical and resource constraints. However, a key strength of topological data analysis is its ability to find patterns in small groups of data^27^. For further studies, increasing the sample size collected will help address most data/performance errors. The inclusion of other cardiac pathologies, such as dilated cardiomyopathy, ischemic cardiomyopathy, and valvular heart diseases, along with the integration of other input data types, such as ratios of regional strains^28^, cardiac MRI, or patient specific demographics, can enhance the versatility of the workflow in the clinical setting. A limitation to the current study is the analysis of only RCM and CP; without consideration of other cardiac pathologies and how their strain parameters may help, or interfere, with correct classification it is unclear how this will generalize to other uncommon pathologies.

The current application of our use-case scenario highlights the ability of TDA, and more specifically persistent homology, to correctly stratify unique cardiovascular anomalies from segmental stress strain analysis.

## Materials and methods

### Study population

In this retrospective case study, we utilized a merged cohort from two previously published datasets^11,12^, with a total of 54 constrictive pericarditis (CP), 49 restrictive cardiomyopathies (RCM), and 55 no structural heart failure control patients (normal).

The institutional review board at the Mayo Foundation approved the protocol outlined by Amaki et al.^11^. Between July 2005 and January 2007, 37 consecutive patients with CP that were scheduled for pericardiectomy treatment and 22 heart failure patients diagnosed with RCM through transthoracic echocardiography; due to suboptimal 2D-echocradiography image quality, seven patients with CP were excluded. Of the remaining patients, 26 with CP and 19 with RCM provided informed consent for participation in the study. Additionally, there was recruitment of 21 control subjects without cardiovascular disease and no evidence of left ventricular dysfunction or significant valvular heart disease observed with echocardiography.

The institutional review board at the Mount Sinai Medical Center approved the protocol by Sengupta et al.^12^. 92 patients (28 with CP, 30 with RCM, and 34 control with no structural/functional abnormality) who underwent transthoracic echocardiography imaging were retrospectively identified.

Three CP patients, 2 RCM patients, and 2 normal patients were excluded from analysis due to incomplete data of all investigated strain values; the remaining dataset utilized in this study had a total of 51 CP, 47 RCM, 53 and control (normal) patients.

### Data security

To maintain confidentiality and integrity of study data, all data was generated, stored, and transmitted using protected encryption measures. Specifically, stored on encrypted drives with industry standard encryption algorithms to prevent unauthorized access. All transmission of data both onsite and offsite was performed using secure protocols to prevent interception and tampering.

### Proposed framework

We propose a persistent homology workflow based on topological data analysis techniques to identify disease patterns from functional physiologic signals. The pipeline is outlined as follows (Fig. 1):

1. Data preprocessing—data is converted to an n-dimensional point cloud.
2. Persistent homology
  - TDA filtration—simplicial complexes are built upon the point cloud, from which topological invariants are extracted.
  - Persistent image—birth, and death features are transformed into a persistent image to develop feature vectors
3. Patient-specific motif—features are stored in a visual representation that can be directly interpreted by physicians/scientists or serve as input for machine learning
  - Direct physician analysis—doctors develop general understanding of underlying patterns visually apparent in motif
  - Machine learning modeling—various techniques applied for feature selection and classification of patients

### Speckle tracking echocardiography

Grayscale images from the apical 4-chamber (4ch) and midventricular parasternal short-axis views were evaluated with 2-dimensional speckle-tracking echocardiography (STE) by a licensed professional as described previously^11,12^. Stress–strain analysis can be segmentally divided into anatomically unique locations, 48 points for short-axis view and 49 points for apical four chamber view, that comprise the entirety of the left ventricular myocardium^29^. At each spatial location, various features, including longitudinal, circumferential, and radial strains and strain rates, were measured over one cardiac cycle. These measurements were stored in two text files, one for the 4ch view and one for the mid view, which serve as the raw data for this proof-of-concept study (Fig. 2).

### Pre-processing

For our purposes, we combined the 48 (short-axis view) and 49 (4ch) segmental strain locations into three functional groupings. The short axis was grouped into the anterior septal, inferior septal, and lateral wall; the apical four chamber view was grouped into the lateral wall, apex, and interventricular septum. Cubic spline interpolation of all strain tracings was performed to standardize patients’ time points within one cardiac cycle. Analysis of segmental strain waveforms as aggregates instead of individual segments has been previously shown^30^. This approach attempts to remove the stochastic nature that analysis of each separate segment would precipitate. Instead, grouping by functional domains allows for averaging curves through a more physiologically relevant manner, specifically regarding the contractile nature and ultrastructural properties of cardiomyocytes within the myocardium^31^. Mean strain curves for each region were created by averaging the corresponding ventricular regions. Each mean strain curve was transformed using phase space reconstruction^32^ using Python library pypsr (v0.0.1) (https://github.com/hsharrison/pypsr) with embedding dimension d = 3 and time delay τ = 2. This reconstruction transformed the echocardiography strain time series into a point cloud in a higher dimensional space (phase space) for subsequent TDA processing, representing a more complete picture of the dynamic system’s linear time signal as a geometric shape, shown in Supplementary Figs. 1 and 2.

### TDA filtration and persistent image

The topological data analysis technique utilized in this study was persistent homology (PH), which is a TDA tool that describes the shape of data by extracting its topological invariants; the mathematical basis of PH is shown in works by Zomorodian and Carlsson^33^, Edelsbrunner et al.^34^, Ghirst et al.^35^, Bubenik et al.^36^ and Adams et al.^37^. The PH concepts relevant to our workflow (Fig. 3) are described briefly in Supplementary File 1.

To accomplish the TDA filtration and conversion to persistent image, we performed this experiment on Python using TDA libraries, ripser (v0.6.0)^38^ and persim (v0.2.0), which are both freely available. To analyze the point clouds, simplicial complex filtration was built on the data points using ripser. The PH of the filtration was extracted as birth and death values for dimension 0. To utilize this information in downstream machine learning tasks, a linear persistent image was created with pixel resolution of [1, 50] and variance of 0.005; the image bounds were automatically selected by the persim algorithm. A total of 18 persistent images each with 50 pixels were created, one for each combination of strain type and wall region; stacking these 18 linear images together generated a specific motif for each patient with a total of 18 by 50 pixels. The feature vector utilized in this study contained the intensities of these 900 persistent image pixels. The computational time for this workflow was approximately 1 min per patient data performed on a MacBook Pro laptop with 2.7 GHz with 16 GB ram without distributed computing.

### Patient-specific motif

Our workflow produced a visual representation indicative of the initial input that can interpreted directly by physicians/scientists while also being capable of feeding into downstream machine learning tasks. The patient-specific motifs showcase the general trends of the disease conditions while maintaining individual patient characteristics, allowing the patients to be monitored for cardiac function changes.

### Statistical analysis

Orange data mining software (v3.28.0) was used for statistical analyses. The models were trained and evaluated using tenfold cross validation. Each model’s receiver operating characteristic (ROC) curve, the area under the curve (AUC), sensitivity, and specificity were calculated to evaluate discriminatory power. To determine the significance of the AUC, a p-value less than 0.05 was considered statistically significant when using the pROC package in R (v4.0.3). To avoid an overfitting problem, Boruta^39^ feature selection was performed through R (v4.0.3) statistical suite that applies a random forest algorithm to determine meaningful features to retain.

### Ethics declarations

All studies were in accordance with the ethical standards of the institutional and national research committee and with the 1964 Helsinki Declaration. For one patient cohort, the institutional review board at Mount Sinai Medical Center approved the protocol, and for second patient cohort, the institutional review board at Mayo Foundation approved the protocol. Participants were included regardless of gender, race, ethnicity, or other demographic factors.

### Supplementary Information

Supplementary Information.

Supplementary Legends.

Supplementary Figure 1.

Supplementary Figure 2.

## Data Availability

The datasets and computer code produced in this study can be provided upon request to the corresponding author(s). A link to the data is provided: https://github.com/qahathaway/TDA_Persistent_Homology.

## Abbreviations

4ch: 4-Chamber
AUC: Area under curve
CP: Constrictive pericarditis
GLS: Global longitudinal strain
PH: Persistent homology
PI: Persistent image
RCM: Restrictive cardiomyopathy
Sn: Sensitivity
Sp: Specificity
STE: Speckle-tracking echocardiography
TDA: Topological data analysis
