# Supplementary material for: A novel multi-task machine learning classifier for rare disease patterning using cardiac strain imaging data
Source: Sci Rep. 2024 May 9;14:10672. doi: 10.1038/s41598-024-61201-4 (PMC11082231; doi:10.1038/s41598-024-61201-4)
Supplement: Supplementary file 1 — Supplementary Information. [file 41598_2024_61201_MOESM1_ESM.docx]

We consider a point cloud data set within a finite metric space, a set M is a function d: M×M to [0, ∞), where [0, ∞) denotes the set of non-negative real numbers.

For any points x,y,z in M,d(x,y)=d(y,x),d(x,x)=0, and d(x,z)≤d(x,y)+d(y,z).

The initial step of PH is a filtration to create a series of simplicial complexes for a scale r, where a sphere of radius r is drawn around every point. At each intersection of two spheres we draw an edge, between the two points. The filtering of data builds a simplicial complex space, from which PH quantifies the presence of n-dimensional holes, i.e. 0-dimensional holes are connected components, 1-dimensional holes are circles/loops/tunnels, and 2-dimensional holes are voids.

For a data set X ⊆ Rn and scale r ≥ 0, the Cech simplicial complex Cech ( X;r) has:

- vertex set X
- finite simplex (x0, x1,..., xk ) when ∩ki=0 B(xi,r) ≠𝝓.

As it is not possible to determine the optimal value for the scale r, the main principle of PH is to progress through all possible values r (0 < r < ∞) to determine how the homology of these components change [17]. For a given filtration of a simplicial complex, we obtain the output (birth, death) barcode intervals, representatives for each topological feature, i.e., for each structure (n-dimensional hole), we compute the times of the birth (at what r it appears), and death (at what r it disappears) (**Figure 3**).

The horizontal axis shows the filtration steps. Each D-dimensional topological feature in filtration is represented by a bar that starts at the filtration step at which the feature is born and ends at the filtration step at which it dies. Thus, for the 0-dimensional barcode, each bar corresponds to a connected component, and the length of a bar indicates how long a particular component remains disconnected from other components.

To compare the homology of persistence diagrams, metrics, such as Wasserstein or Bottleneck distance, can be calculated. Persistence barcodes shown by Ghirst [19] and persistence landscapes shown by Bubenik et al. [20] have been developed to represent the persistent topology within datasets. Adams et al. have introduced persistence images to vectorize persistence diagrams for machine learning tasks [21]. We have selected the persistence image methodology because of its ability to work with a broader range of machine learning algorithms and the potential to convert its feature vectors into a visual patient motif. The persistence image (PI) pipeline converts the birth-death points to birth-persistence, i.e. (b, d) to (b, d-b). A weighting function is applied, giving points that are more persistent a higher amount of intensity. Gaussian probability distribution with selected variance level is applied at each point. A grid “n” by “n” is overlaid over the surface to form the PI with chosen resolution. The pixel intensities of the PI are taken as a feature vector for machine learning and feature selection. Vectors from different component dimensions can be concatenated into a disease pattern motif for both visualization and storage.

**Figure 3:**

**
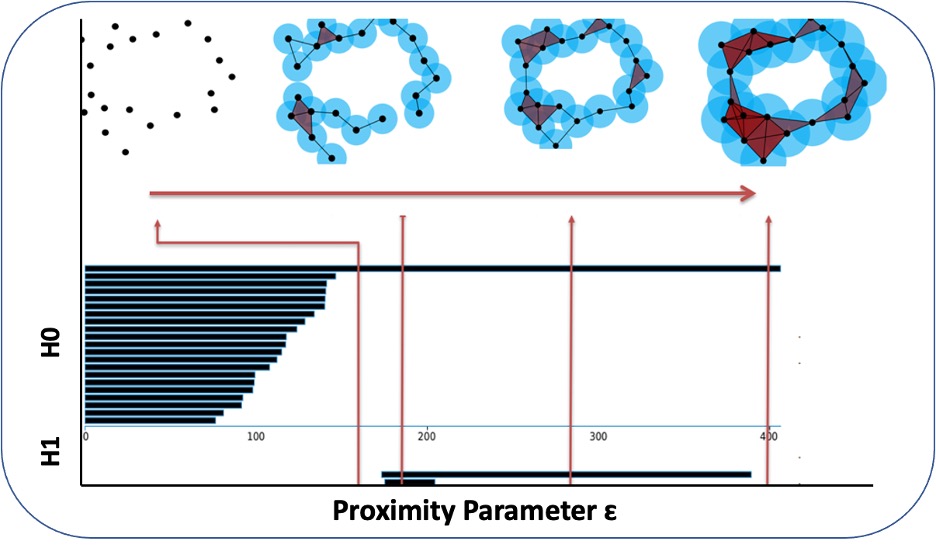
**

**Persistent Homology Explanation.** For a given proximity parameter ε, a circle with radius = ε is drawn around each data point. The intersections of these circles guide the construction of a set of simplicial complexes. All possible values of ε are tested to detect variations in topology at different scales. The appearance and disappearance of connected components and open loops is measured by H0 and H1, respectively, and subsequently visualized as a persistent barcode. H0 represents dimension 0 persistent homology, and H1 represents dimension 1 persistent homology.

**References:**

19 Ghrist R (2008) Barcodes: The persistent topology of data. Bulletin of the American Mathematical Society 45:61-75

20 Bubenik P (2015) Statistical Topological Data Analysis using Persistence Landscapes. Journal of Machine Learning Research 16:77−102

21 Adams H, Emerson T, Kirby M et al (2017) Persistence Images: A Stable Vector Representation of Persistent Homology. Journal of Machine Learning Research 18:1−35
