# Supplementary material for: A novel multi-task machine learning classifier for rare disease patterning using cardiac strain imaging data
Source: Sci Rep. 2024 May 9;14:10672. doi: 10.1038/s41598-024-61201-4 (PMC11082231; doi:10.1038/s41598-024-61201-4)
Supplement: Supplementary file 2 — Supplementary Legends. [file 41598_2024_61201_MOESM2_ESM.docx]

**Supplementary Figure 1**: **Original phase reconstruction point clouds for septal longitudinal strain analysis.** Plots represent data prior to persistent homology filtration step. Restrictive cardiomyopathy (RCM) patients have point trajectory that does not disperse as much as constrictive pericarditis (CP) and normal patients (NL); this is representative of the restriction in longitudinal strain in this wall region. This information is captured in the patient specific motif by RCM patients having decreased intensity in the higher pixel values of septal longitudinal strain compared to the other groups.

**Supplementary Figure 2:** **Original phase reconstruction point clouds for apical radial strain analysis.** Plots represent data prior to persistent homology filtration step. Constrictive pericarditis (CP) patients have point trajectory that disperses more than as restrictive cardiomyopathy (RCM) and normal (NL) patients; this is representative of the enhanced apical strain in this wall region. This information is captured in the patient specific motif by CP patients having increased intensity in the higher pixel values of apical radial strain compared to the other groups.
