# Supplementary figures and images for: A novel multi-task machine learning classifier for rare disease patterning using cardiac strain imaging data
Source: Sci Rep. 2024 May 9;14:10672. doi: 10.1038/s41598-024-61201-4 (PMC11082231; doi:10.1038/s41598-024-61201-4)

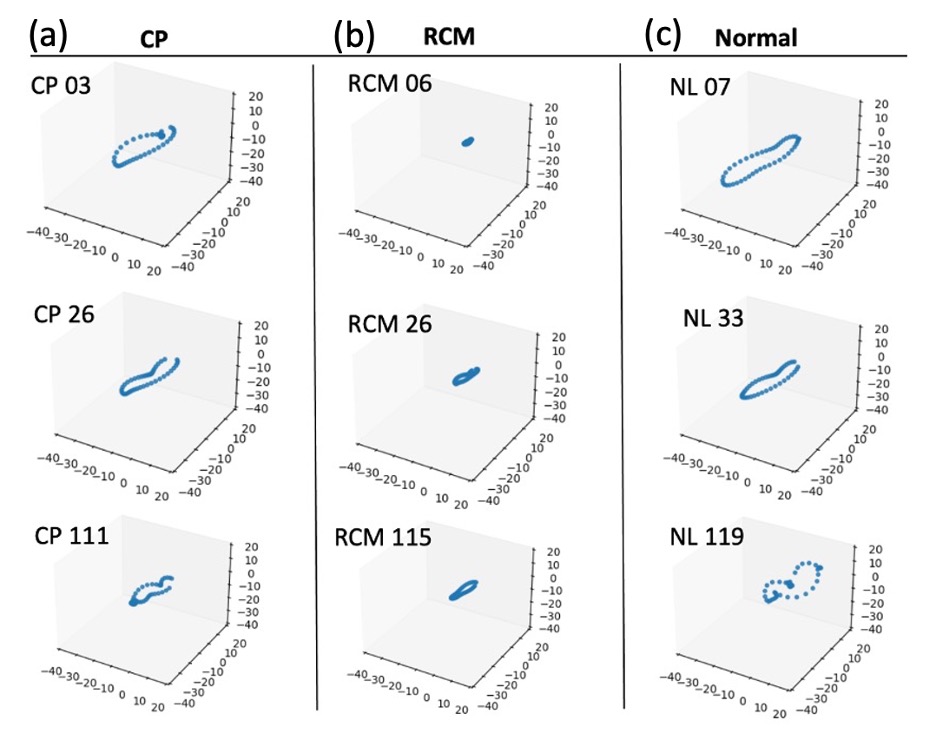

Supplement: Supplementary file 3 — Supplementary Figure 1. [file 41598_2024_61201_MOESM3_ESM.jpg]

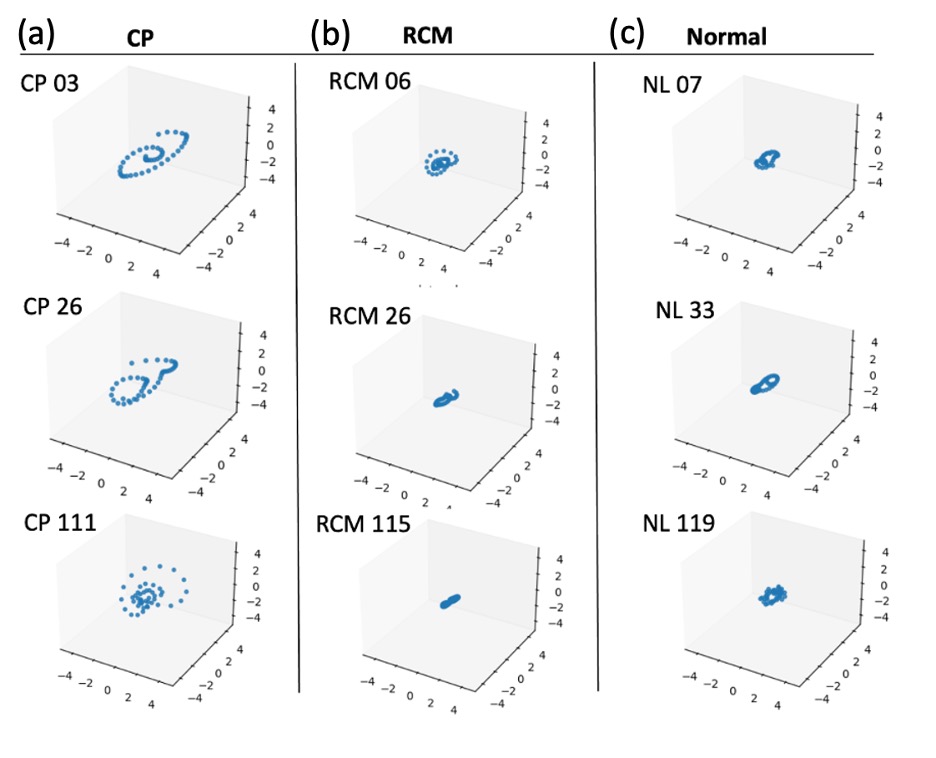

Supplement: Supplementary file 4 — Supplementary Figure 2. [file 41598_2024_61201_MOESM4_ESM.jpg]
